# Differential regulation of riboflavin supply genes in *Vibrio cholerae*

**DOI:** 10.1186/s13099-017-0159-z

**Published:** 2017-02-15

**Authors:** Ignacio Sepúlveda Cisternas, Alexia Torres, Andrés Fuentes Flores, Víctor Antonio García Angulo

**Affiliations:** 1 Escuela de Biotecnología, Universidad Mayor, Campus Huechuraba, Santiago, Chile; 2 Programa de Microbiología y Micología, Instituto de Ciencias Biomédicas, Facultad de Medicina Norte, Universidad de Chile, Pabellón L. Independencia, 1027, 8380453 Santiago, Chile

**Keywords:** *Vibrio cholerae*, Riboflavin, Transporter, Biosynthesis, Gene regulation

## Abstract

**Background:**

Riboflavin is the precursor of important redox cofactors such as flavin mononucleotide (FMN) and flavin adenine dinucleotide, required for several biological processes. *Vibrio cholerae*, a pathogenic bacterium responsible for the cholera disease, possesses the ability to biosynthesize de novo as well as to uptake riboflavin through the riboflavin biosynthetic pathway (RBP) and the RibN importer, respectively. The intra-organism relationship between riboflavin biosynthesis and uptake functions has not been studied.

**Results:**

This work determined the transcriptional organization of RBP genes and *ribN* in *V. cholerae* through reverse transcription polymerase chain reaction and analyzed their expression when growing with or without extracellular riboflavin using real time PCR. The RBP is organized in three transcriptional units, the major one containing *ribD, ribE, ribA* and *ribH* together with genes involved in functions not directly related to riboflavin biosynthesis such as *nrdR* and *nusB*. In addition, two independent monocistronic units contain *ribA2* and *ribB*, the later conserving a putative FMN riboswitch. The *ribN* gene is encoded in operon with a gene coding for a predicted outer membrane protein and a gene encoding a protein with a glutaredoxin domain. Regulation analysis showed that among these transcriptional units, only *ribB* is negatively regulated by riboflavin and that its repression depends on the RibN riboflavin importer. Moreover, external riboflavin highly induced *ribB* transcription in a *ΔribN* strain. Also, a genomic database search found a negative correlation between the presence of *nrdR* and *nusB* and the FMN riboswitch in bacterial RBP operons.

**Conclusions:**

Growing in the presence of riboflavin downregulates only a single element among the transcriptional units of riboflavin supply pathways. Thus, endogenous riboflavin biosynthesis seems to be negatively regulated by extracellular riboflavin through its specific effect on transcription of *ribB* in *V. cholerae*.

**Electronic supplementary material:**

The online version of this article (doi:10.1186/s13099-017-0159-z) contains supplementary material, which is available to authorized users.

## Background

Flavin mononucleotide (FMN) and flavin adenine dinucleotide (FAD), the main derivatives of riboflavin, are cofactors for enzymes mediating many redox reactions in the cell [1]. Globally, up to 17% of enzymes dependent on cofactors use flavins [2]. Flavoenzymes are involved in a mixture of biological processes such as vitamin, fat and carbohydrate metabolism, photosensitization and oxidative stress response. In bacteria, flavins *per se* are also involved in extracellular processes such as assimilatory iron reduction, extracellular respiratory chain and symbiotic interactions [1, 3–6].

There are two ways bacteria can obtain riboflavin, one of them being the riboflavin biosynthetic pathway (RBP) and the other the riboflavin importer systems. The RBP uses guanosine-5-triphosphate (GTP) and ribulose 5-phosphate as precursors for the biosynthesis of riboflavin. Generally, the RBP consists of five enzymes, GTP cyclohydrolase II (RibA), a bifunctional pyrimidine deaminase/reductase (RibD), 3,4-dihydroxy-2-butanone-4-phosphate (3,4-DHBP) synthase (RibB), 6,7-dimethyl-8-ribityllumazine (lumazine) synthase (RibH) and riboflavin synthase (RibE) [1, 7, 8]. To date, nine riboflavin transport systems have been identified in bacteria. This include the energy-coupling factor system-RibU, RfuABCD and RibXYZ, which are members of the ATP binding cassette family of transporters, and the RfnT, RibM, RibN, RibZ and RibV transporters [9–17]. The transcriptional organization of the RBP genes largely differ among bacteria species. While some species arrange the full pathway genes in a single operon, others have the genes scattered in different transcriptional units along the chromosome. In addition, operons with RBP genes may also contain genes not directly related to riboflavin biosynthesis. Particularly, the global regulators *nrdR* and *nusB*, encoding a transcriptional repressor of nucleotide reductases and other genes and a factor of the bacterial antiterminator complex, respectively, have been found genetically associated to RBP in various species [14, 16, 18–22]. In some bacteria, RBP and riboflavin importer genes conserve the FMN riboswitch [9, 12, 15, 16, 23–25]. This is a genetic element encoded in RNA leader regions, which downregulates transcription and/or translation by adopting alternative expression-permissive or expression-repressive secondary structures in response to FMN binding [25, 26]. Thus, many riboflavin supply genes seem to be regulated in response to intracellular flavin levels.

Despite the fact that some bacteria lack the RBP and fulfill their riboflavin demands through riboflavin uptake, in many organisms the RBP and riboflavin importer genes coexist [10, 15, 27, 28]. Recently, a search on a set of fully sequenced bacterial genomes showed that most bacteria with a riboflavin importer also encode the RBP. Moreover, some species conserve two different families of riboflavin importers besides the RBP [12]. Additionally, bacteria may encode duplicated or multiplicated orthologs of some RBP enzymes. The complexity of bacterial riboflavin supply pathways seem to respond to species-specific riboflavin needs. For example, a duplicated RibH ortholog in *Brucella abortus* is specifically associated to survival inside the host and the *ribBA* gene of *Sinorhizobium meliloti* is specialized in the production of riboflavin targeted for secretion [29, 30]. This has led to the hypothesis that riboflavin biosynthesis has a modular structure in bacteria [30]. Although it is possible that riboflavin importers substitute for the RBP in riboflavin prototrophs when environmental riboflavin is available, the way intraspecies riboflavin provision pathways coordinate to accomplish flavin requirements in bacteria has been scarcely studied.

*Vibrio cholerae* is an aquatic gammaproteobacteria that causes cholera, a human pandemic disease characterized by acute watery diarrhea, which can lead to death in a short term if untreated. Normally thriving in sea and estuarine waters, the environmental cycle of *V. cholerae* includes biofilm formation in biotic and abiotic surfaces and the entrance into the metabolically quiescent viable but non-culturable state under unfavorable conditions [31]. After human consumption, *V. cholera* expresses a series of virulence factors in human intestinal tract, most notably the cholera toxin and the toxin-coregulated pilus. These factors contribute to host colonization and diarrhea development [32].

*Vibrio cholerae* is a riboflavin prototroph and it also has the ability to scavenge riboflavin through the RibN riboflavin importer [14, 33]. Given the wide range of conditions comprising its life cycle, it is likely that *V. cholerae* faces variable riboflavin concentrations. This feature makes it an interesting species to study the interconnections between riboflavin biosynthesis and uptake. The present work determined the transcriptional organization of the RBP and *ribN* genes in *V. cholerae*. In addition, to gain insights into the cues governing the interrelation between riboflavin biosynthesis and transport, we investigated the effect of extracellular riboflavin on the expression of the transcriptional units encoding the riboflavin supply pathways.

## Results

### Transcriptional organization of riboflavin supply pathways in *V. cholerae*

**Fig. 1:**
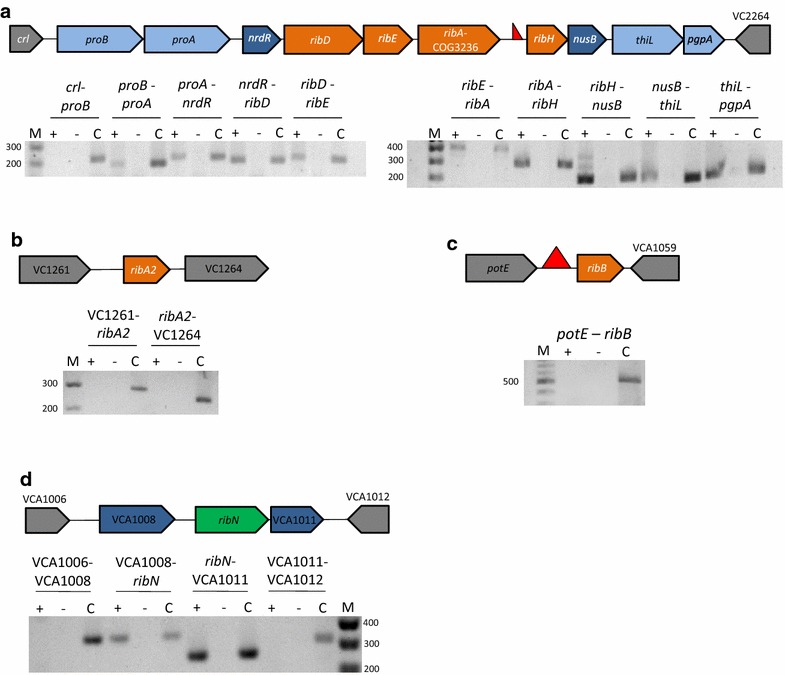
Assessment of the transcriptional organization of *rib* genes in *V. cholerae*. PCR reactions were carried on cDNA from *V. cholerae* with primers designed to amplify the indicated gene junctions of *rib* genes and their flanking genes in the loci encoding *ribD*, *ribE*, *ribA*-COG3236 and *ribH* (**a**), *ribA2* (**b**), *ribB* (**c**) and *ribN* (**d**). Each reaction was independently performed three times with the same results. *+* Template cDNA derived from RT-PCR with reverse transcriptase, *−* template cDNA derived from RT-PCR without reverse transcriptase (negative control), *C* PCR with chromosomal DNA as template (positive control), *M* molecular weight marker (basepairs). *Isosceles triangle* over the regulatory region indicates FMN riboswitch. *Half Isosceles triangle* indicates truncated FMN riboswitch

We searched the genome of *V. cholerae* N16961 in the Kyoto encyclopedia of genes and genomes (KEGG) [34] for RBP genes and *ribN*. Results show that this strain conserves a cluster of contiguous *ribD*, *ribE*, *ribA*-COG3236 and *ribH* genes in chromosome 1 (Fig. 1a). This cluster is localized between *nrdR* and *nusB*. The *ribA*-COG3236 gene fusion encodes a protein with GTP cyclohydrolase II (RibA) activity fused to a protein domain belonging to the cluster of orthologous groups (COG) 3236. COG3236 modifies early riboflavin biosynthesis intermediates, cleaving *N*-glycosidic bonds presumably to diminish their reactivity when overproduced. The COG3236 is often found fused to RBP enzymes in plants and bacteria [35]. An alignment performed by us determined that the *V. cholerae* fusion shares 89% identity with the experimentally characterized ortholog in *Vibrio vulnificus*. Importantly, the intergenic region between *ribA*-COG3236 and *ribH* is relatively large (264 bp) and conserves an apparently incomplete FMN riboswitch, as detected by the RIBEX software [36]. Thus, it is not possible to predict hiterto if these genes form an operon. Genes *ribB* and a second ortholog of *ribA*, hereafter referred to as *ribA2*, were identified in different regions of chromosome 2 and 1, respectively. The *ribB* gene conserves an FMN riboswitch. Overall, the distribution and assortment of RBP genes in *V. cholerae* resembles the predicted distribution of such genes in *V. parahaemolyticus* [22]. In addition, the *ribN* gene is localized in chromosome 2 between the VCA1008 and VCA1011 open reading frames, encoding a putative outer membrane protein and a protein containing a glutaredoxin domain, respectively.

To experimentally determine the transcriptional organization of the identified riboflavin supply genes, a reverse transcriptase polymerase chain reaction (RT-PCR) analysis was performed. This carried PCR reactions on complementary DNA (cDNA) obtained from *V. cholerae* RNA with primers designed to amplify putative gene junctions. In this approach, positive PCR amplifications indicate the joint of coding sequences in the same messenger RNA. Results showed that the main RBP operon extends from *proB* to *pgpA*, also including the genes *proA, nrdR, ribD*, *ribE, ribA*-COG3236*, ribH, nusB* and *thiL*. All of the RT-PCR reactions produced amplicons of the expected size for these gene junctions, while negative controls, consisting on templates obtained without reverse transcriptase, did not yield amplicons. Positive controls, using chromosomal DNA as template, yielded amplicons in all cases, including junctions of genes not forming operon (Fig. 1a). A similar analysis showed that *ribA2* and *ribB* genes comprise monocistronic untis (Fig. 1b, c). Likewise, results showed that *ribN* forms an operon with VCA1008 and VCA1011 (Fig. 1d).

### Effect of riboflavin on the expression of riboflavin supply genes

**Fig. 2:**
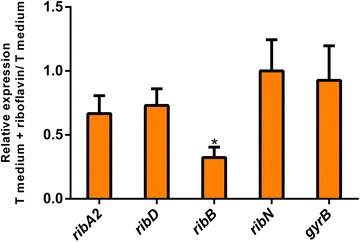
Relative expression of riboflavin supply pathways genes in *V. cholerae* growing in media with and without riboflavin. *V. cholerae* was grown in T minimal media and relative expression of the *ribD*, *ribA2*, *ribB*, *ribN* and *gyrB* genes in cultures with and without added riboflavin was determined by real time PCR. Media and standard deviation from three independent experiments are shown. * Statistical difference compared to the *gyrB* control (p < 0.05) using analysis of variance and Tukey HSD post hoc test

**Fig. 3:**
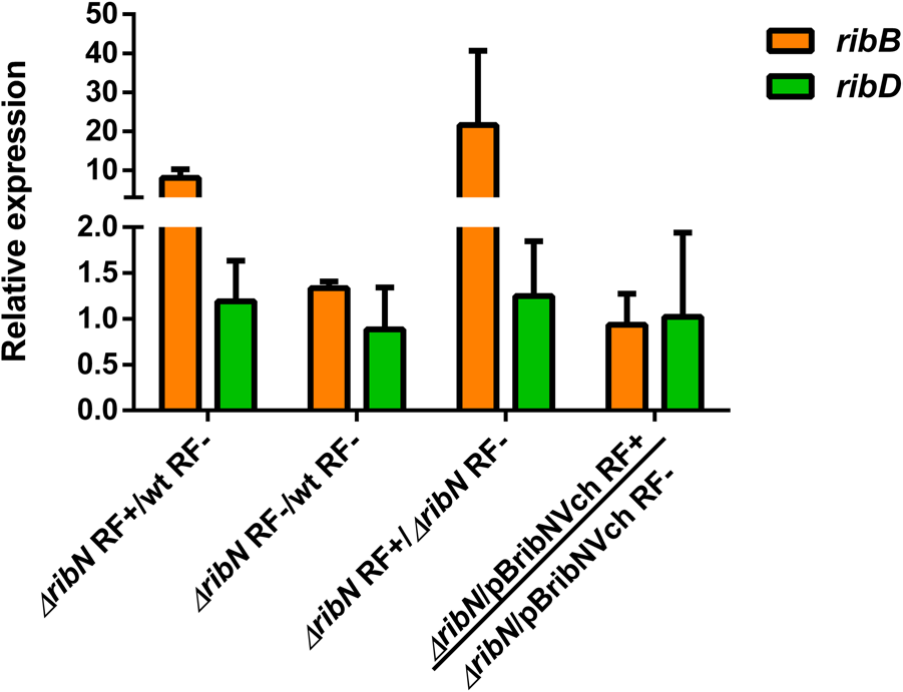
Effect of *ribN* deletion on expression of *ribB* and *ribD.* Relative *ribB* and *ribD* expression in ∆*ribN* with riboflavin and WT without riboflavin, both strains without riboflavin and ∆*ribN* and its complemented derivative growing with or without riboflavin as indicated, determined by real time PCR. Media and standard deviation from three independent experiments are shown. *RF* riboflavin

We assessed the effect of extracellular riboflavin on the expression of the four transcriptional units identified. Bacteria were grown in T minimal medium with or without riboflavin and the relative expression among these two conditions of *ribD*, *ribA2*, *ribB* and *ribN* was assessed by real time PCR (Fig. 2). This assay also included the housekeeping *gyrB* gene unrelated to riboflavin provision as a control. Results showed that *ribB* transcription was reduced by two-thirds when growing in the presence of riboflavin. Although in this experiment the expression of *ribD* and *ribA2* was somewhat reduced in the presence of riboflavin, this effect was not statistically significant. Meanwhile, *ribN* expression was not affected. To determine if the negative regulatory effect of riboflavin on *ribB* depends on the activity of the RibN riboflavin importer, we constructed a *ribN* null mutant and assessed its *ribB* expression. Unexpectedly, the elimination of *ribN* caused a sevenfold increase in the expression of *ribB* when growing in the presence of riboflavin when compared to the WT without riboflavin (Fig. 3). This overexpression is indeed dependent on extracellular riboflavin, as the expression of *ribB* in the *∆ribN* mutant is similar to that of the WT when no riboflavin is added to the medium. Moreover, the expression of *ribB* on the *∆ribN* is increased by 19 fold by the presence of riboflavin. This overexpression was prevented by the complementation of the *∆ribN* strain with a plasmid encoding *ribN* (pBribNVch). In all cases, *ribD* expression remained around the same irrespective of the presence of riboflavin. These results show not only that RibN is necessary to repress *ribB* in response to riboflavin, but that extracellular riboflavin highly induces the expression of this gene when the RibN riboflavin importer is absent.

### The presence of the FMN riboswitch negatively correlates with *nrdR* and *nusB* in bacterial RBP operons

The expression profiles defined here suggest that only *ribB* is significantly downregulated in response to extracellular riboflavin. The presence of genes involved in functions different to riboflavin biosynthesis in the main operon, most notably the *nrdR* and *nusB* global regulators, may preclude its systematic regulation by riboflavin levels. As association of FMN riboswitch and *nrdR* and *nusB* to RBP genes is widespread in bacteria [12, 16, 37, 38], we seek to estimate the co-occurrence of these genetics elements in RBP genes. For this purpose, we searched the bacterial genomes in the *P*rokaryotic *O*peron *D*ata *B*ase (*P*ro*O*p*DB*) for the presence of the FMN riboswitch. *P*ro*O*p*DB* contains the predicted transcriptional organization of a set of fully sequenced prokaryotic genomes [39]. 651 predicted bacterial transcriptional units displayed by the *P*ro*O*p*DB* contained the FMN riboswitch (element RF 00050) (Additional file 1). While almost all of them clustered RBP and/or riboflavin importer genes, none of them included *nrdR* and only five of them included *nusB*. On the other side, a search for the transcriptional organization of *nrdR* homologues in the database shows its association with RBP genes in 86 transcriptional units, none of them conserving the FMN element (Additional file 2). Overall, *P*ro*O*p*DB* search results support the hypothesis that riboflavin biosynthesis is not repressed by flavins through the FMN riboswitch when the operon encodes additional genes not directly related to riboflavin biosynthesis such as the global regulators NrdR and NusB.

## Discussion

Results showed that riboflavin provision pathways in *V. cholerae* are encoded in four transcriptional elements. The main RBP operon includes the genes *ribD*, *ribE*, *ribA*-COG3236 and *ribH*, together with genes involved in proline biosynthesis (*proBA*), thiamin biosynthesis (*thiL*) and a phosphatidylglycerophosphatase (*pgpA*) in addition to *nusB* and *nrdR*. In spite of its large upstream intergenic region, *ribH* and downstream genes are part of the operon. Noteworthy, this region conserves a truncated riboswitch. This may hint to a relatively recent integration of this segment to the operon. Two other RBP components, *ribB* and *ribA2* were found to be encoded monocistronically. The conservation of a FMN riboswitch only in *ribB* suggested a differential regulation for the RBP components *a priori*. Later results showed a negative correlation between *nrdR* and *nusB* and the FMN riboswitch in RBP genes. This is an interesting finding as it starts to define patterns in the apparently highly variable ways bacteria transcriptionally arrange their RBP genes [28]. Finally, RibN was found encoded in operon with a putative outer membrane protein (VCA1008) and a protein with a domain of unknown function fused to a glutaredoxin-like domain (VCA1011). Glutaredoxins are enzymes involved in redox homeostasis in bacteria [40]. Thus, it is possible that imported riboflavin is related to functions of VCA1008 and VCA1011.

Riboflavin needs in bacteria are highly diverse and seem to be quite dependent on species specific traits. While some bacteria may fulfill all of its riboflavin needs through the RBP and others rely solely on riboflavin uptake, many have conserved both functions. The selection constraints driving this phenomenon are not known. It has been pointed out that riboflavin biosynthesis requires more metabolic energy than its transport inside the cell [41]. Hence, activation of riboflavin uptake and biosynthesis halt when growing in nutrient rich environments would help save energy in a prototroph species. This could be the case for *V. cholerae*. Growing on riboflavin rich media downregulated *ribB*. Noteworthy, RibB activity is the only one lacking on the otherwise full RBP encoded in the main riboflavin biosynthetic operon. Thus, it seems that the extracellular availability of riboflavin diminishes endogenous riboflavin biosynthesis at the level of 3,4-DHBP synthesis. Nonetheless, *ribB* transcription was not completely abolished. This may reflect that some *ribB* expression is still afforded in the presence of extracellular riboflavin or that the *ribB* FMN riboswitch also acts at the translational level to fully shut down expression, as recently shown for the FMN riboswitch in *ribB* from *Escherichia coli* [25]. It is intriguing why constitutive expression of the rest of the RBP enzymes is maintained in the presence of extracellular riboflavin. One explanation for this may be the participation of these enzymes in other metabolic pathways. Indeed, RibA has been involved in folate biosynthesis in *Chlamydia trachomatis* [42] and it has been suggested a participation of RibD and RibA in toxoflavin biosynthesis in *Pseudomonas protegens* [43].

RibN was necessary for external riboflavin to repress *ribB*. Moreover, extracellular riboflavin highly induced the expression of *ribB* after deletion of *ribN*. Importantly, this result may imply that the presence of extracellular riboflavin triggers riboflavin-dependent traits inside the cell. In WT strain, these emergent riboflavin demands may be fulfilled through the RibN uptake activity. However, the ∆*ribN* strain may suffer a high reduction of intracellular flavin levels in such conditions, which could account for *ribB* overexpression.

Overall, results indicate that *V. cholerae* riboflavin provision pathways components are encoded in four transcriptional units. These units are differentially regulated by riboflavin, which may putatively reduce riboflavin biosynthesis through repression of RibB transcription. Although the transcription of the *ribN* riboflavin importer gene was not affected, the presence of riboflavin may trigger responses that increase the intracellular flavin requirements. Further experimental work is needed to effectively determine if endogenous riboflavin biosynthesis is diminished when growing in the presence of riboflavin, and to assess the bacterial physiological traits activated in response to it.

## Methods

### Growth conditions, RNA extraction, retrotranscription and PCR analysis

*Vibrio cholerae* N16961 strain and its ∆*ribN* derivative were grown overnight in LB plates at 37 °C. 5 ml of LB broth were inoculated with these overnight cultures and incubated at 37 °C in an orbital shaker at 150 rpm until they reached an OD_600 nm_ of 1.0. Next, cultures were centrifuged, supernatant discarded, pellet washed two times with T minimal medium [44] and resuspended in 1 ml of fresh T medium. 10 µl of these resuspensions were used to inoculate 10 ml of T medium and T medium plus 2 µM riboflavin. Cultures were incubated at 37 °C with 180 rpm shaking until an OD_600 nm_ of 0.8. Next, 3 ml of these cultures were pelleted and subjected to RNA extraction using the Thermo Scientific Genejet RNA purification kit according to manufacturer´s instructions. RNA extracts were digested with Turbo DNA-free DNAase at 37 °C for 1 h. cDNA synthesis was performed with AffinityScript QPCR cDNA Synthesis kit (Agilent Technologies) according to manufacturer’s instructions. A control reaction with no reverse transcriptase was included for each sample. PCR reactions using primers designed to amplify gene junctions were performed on these cDNA. Primers’ sequences are detailed in Additional file 3.

### Real time PCR

Real time PCR assays were performed using cDNA obtained as previously described as templates and using the Brilliant II SYBR Green QPCR Master Mix kit in a One-Step Applen Biosystems (Life Technologies) thermocycler. Relative expression was calculated using the ∆∆Ct method as developed by Livak et al. [45]. 16s RNA subunit gene was used for normalization. Primers used for each gene for the real time PCR assay are detailed in Additional file 3.

### Construction of *ribN* mutant and complementation plasmid

Deletion of *ribN* in *Vibrio cholerae* was performed with homologous recombination using PCR products as reported before [46]. Briefly, WT strain was transformed with plasmid pKD46 encoding λ phage Red recombinase system. Next, a PCR product containing a kanamycin resistant cassette flanked by sites homologous to *ribN* 5′ and 3′ ends, using primers ribNH1P1and ribNH2P2 (Additional file 3) and plasmid pKD4 as template was obtained. The PCR product was electroporated into WT strain carrying pKD46, recombination system expression induced with arabinose and recombinants selected in LB+kanamycin plates. Positive clones were tested for the replacement of *ribN* by the kanamycin cassette by PCR using primers VCA1008FwHindIII and grxRv, flanking the insertion site. One of the positive clones was electroporated with plasmid pCP20, encoding FRT recombinase. After growth in nonselective LB media, clones were analyzed for the loss of the kanamycin cassette by testing antibiotic sensibility and PCR using primers VCA1008FwHindIII and grxRv. To construct a complementation plasmid with *ribN*, this gene was amplified from *V. cholerae* genomic DNA with PCR using primers VCA1008FwHindIII and ribNRvFLAGPstI. The resulting product was cloned in the pGEM T Easy commercial cloning vector (Promega). Next, *ribN* was excised and subcloned into the PstI/XbaI sites of pBBR1 MCS 1 vector. This rendered the pBribNVch plasmid. The loss of expression of *ribN* transcript in the *∆ribN* strain as well as the rescue of expression by the pBribNVch plasmid were corroborated by end point RT-PCR with primers ribNFw and ribNRv (Additional file 4).

## Additional files

**Additional file 1.** *P*ro*O*p*DB* output for the search of the FMN riboswitch (RF00050), visualized with GeConT [47]. Top of the figure shows the putative transcriptional organization of elements containing the FMN riboswitch in bacterial genomes. Bottom lists the clusters of orthologous groups (COG) contained in genes depicted in the top. Note that the COG1327, clustering *nrdR* homologs, is absent from the list.

**Additional file 2.** *P*ro*O*p*DB* output for the search of the COG1327 (*nrdR*), visualized with GeConT. Top of the figure shows the putative organization of transcriptional units containing the COG1327. Bottom lists the COGs contained in genes depicted in the top.

**Additional file 3.** List of primers used in this study.

**Additional file 4.** Lack of expression of *ribN* in the *V. cholerae ∆ribN* strain and complementation by the pBribNVch plasmid. The presence of the *ribN* messenger RNA was assessed by end-point real time PCR using primers ribNFw and ribNRv and cDNA from the indicated strains. +, template cDNA derived from RT-PCR with reverse transcriptase; −, template cDNA derived from RT-PCR without reverse transcriptase (negative control); C, PCR with genomic DNA as template (positive control).
