# Supplementary figures and images for: Differential regulation of riboflavin supply genes in Vibrio cholerae
Source: Gut Pathog. 2017 Feb 15;9:10. doi: 10.1186/s13099-017-0159-z (PMC5312566; doi:10.1186/s13099-017-0159-z)

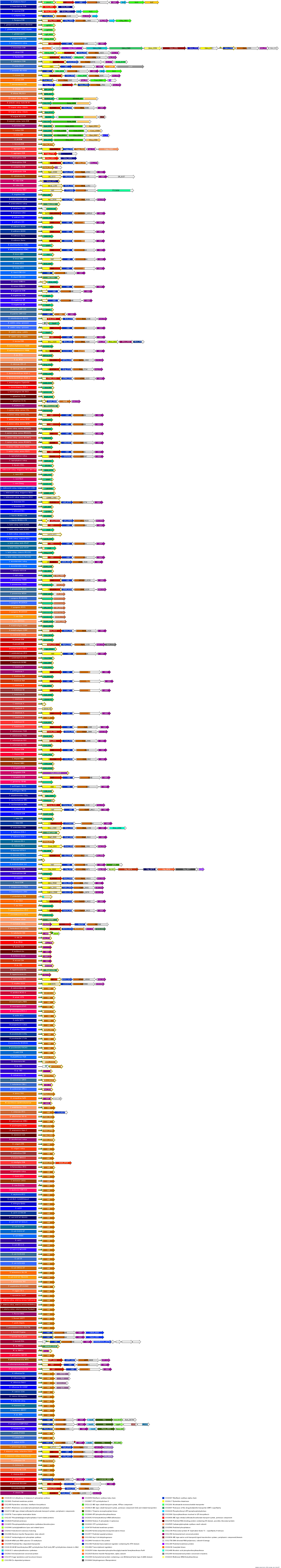

Supplement: Supplementary file 1 — Additional file 1. ProOpDB output for the search of the FMN riboswitch (RF00050), visualized with GeConT [47]. Top of the figure shows the putative transcriptional organization of elements containing the FMN riboswitch in bacterial genomes. Bottom lists the clusters of orthologous groups (COG) contained in genes depicted in the top. Note that the COG1327, clustering nrdR homologs, is absent from the list. [file 13099_2017_159_MOESM1_ESM.png]

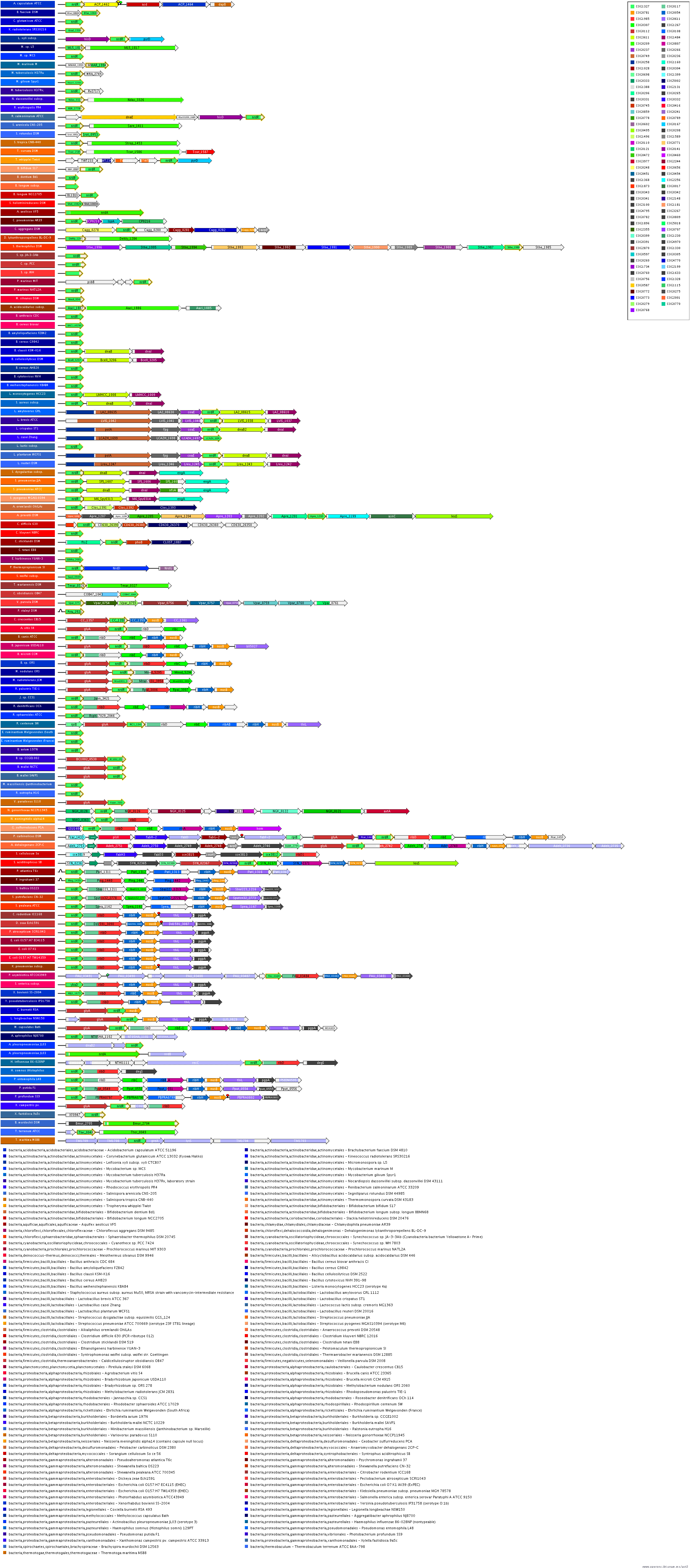

Supplement: Supplementary file 2 — Additional file 2. ProOpDB output for the search of the COG1327 (nrdR), visualized with GeConT. Top of the figure shows the putative organization of transcriptional units containing the COG1327. Bottom lists the COGs contained in genes depicted in the top. [file 13099_2017_159_MOESM2_ESM.png]

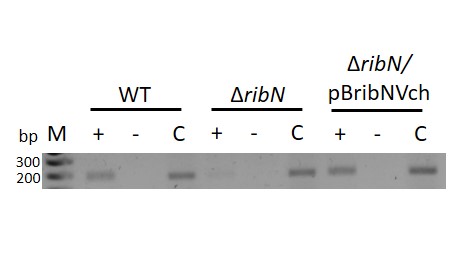

Supplement: Supplementary file 4 — Additional file 4. Lack of expression of ribN in the V. cholerae ∆ribN strain and complementation by the pBribNVch plasmid. The presence of the ribN messenger RNA was assessed by end-point real time PCR using primers ribNFw and ribNRv and cDNA from the indicated strains. +, template cDNA derived from RT-PCR with reverse transcriptase; −, template cDNA derived from RT-PCR without reverse transcriptase (negative control); C, PCR with genomic DNA as template (positive control). [file 13099_2017_159_MOESM4_ESM.jpg]
